# Modulation of microglial activation states by spinal cord stimulation in an animal model of neuropathic pain: Comparing high rate, low rate, and differential target multiplexed programming

**DOI:** 10.1177/1744806921999013

**Published:** 2021-02-24

**Authors:** William J Smith, David L Cedeño, Samuel M Thomas, Courtney A Kelley, Francesco Vetri, Ricardo Vallejo

**Affiliations:** 1 Research and Development, Lumbrera LLC, Bloomington, IL, USA; 2 Geisel School of Medicine, Dartmouth College, Hanover, NH, USA; 3 Department of Psychology, Illinois Wesleyan University, Bloomington, IL, USA; 4 College of Osteopathic Medicine, Des Moines University, Des Moines, IA, USA; 5 National Spine and Pain Centers, Bloomington, IL, USA

**Keywords:** spinal cord stimulation, differential target multiplexed (DTM) programming, chronic neuropathic pain, microglia transcriptome

## Abstract

While numerous studies and patient experiences have demonstrated the efficacy of spinal cord stimulation as a treatment for chronic neuropathic pain, the exact mechanism underlying this therapy is still uncertain. Recent studies highlighting the importance of microglial cells in chronic pain and characterizing microglial activation transcriptomes have created a focus on microglia in pain research. Our group has investigated the modulation of gene expression in neurons and glial cells after spinal cord stimulation (SCS), specifically focusing on transcriptomic changes induced by varying SCS stimulation parameters. Previous work showed that, in rodents subjected to the spared nerve injury (SNI) model of neuropathic pain, a differential target multiplexed programming (DTMP) approach provided significantly better relief of pain-like behavior compared to high rate (HRP) and low rate programming (LRP). While these studies demonstrated the importance of transcriptomic changes in SCS mechanism of action, they did not specifically address the role of SCS in microglial activation. The data presented herein utilizes microglia-specific activation transcriptomes to further understand how an SNI model of chronic pain and subsequent continuous SCS treatment with either DTMP, HRP, or LRP affects microglial activation. Genes for each activation transcriptome were identified within our dataset and gene expression levels were compared with that of healthy animals, naïve to injury and interventional procedures. Pearson correlations indicated that DTMP yields the highest significant correlations to expression levels found in the healthy animals across all microglial activation transcriptomes. In contrast, HRP or LRP yielded weak or very weak correlations for these transcriptomes. This work demonstrates that chronic pain and subsequent SCS treatments can modulate microglial activation transcriptomes, supporting previous research on microglia in chronic pain. Furthermore, this study provides evidence that DTMP is more effective than HRP and LRP at modulating microglial transcriptomes, offering potential insight into the therapeutic efficacy of DTMP.

## Introduction

Chronic pain represents a sizable burden to the United States healthcare system, affecting approximately 7–10% of the population.^1^ As such, considerable research efforts are aimed at understanding the development and maintenance of chronic pain, with hopes that improved understanding begets improved therapeutic options. One such area of research focuses on the role of glial cells in neuropathic pain and the effect of electrical neuromodulation therapies, such as spinal cord stimulation (SCS), on the interactions between these cells and neurons.

Although neurons and glial cells are the main cellular constituents of neural tissue, glial cells are more abundant than neurons in the spinal cord of primates, including humans.^2–4^ Among spinal glia, microglia and astrocytes are known to be integral to the establishment and maintenance of neuropathic pain.^5–7^ Our group previously reported on the distinctive response of glia and neurons when exposed to different electrical signals,^8,9^ demonstrating that electrical stimulation of the spinal cord may be used to differentially modulate those cells and provide improved relief of neuropathic pain in an animal model. Despite the established importance of glial cells in neuropathic pain, traditional models of mechanisms of action of SCS have largely neglected their multifactorial role in neuronal modulation. Glial cells are key players in the so-called “tripartite synapse” and contribute to determine the modifications of synaptic structure and function as well as excitation/inhibition homeostasis via network and molecular changes.^10,11^ Recently, our lab and others have shown, using transcriptomics and proteomics in animal models, that neuropathic pain induces a complex response composed of metabolic, inflammatory, and immune biological processes involved in neuron-glial interactions that SCS can modulate.^8,9,12,13^

Spinal microglia are known to undergo profound changes upon activation by a number of stimuli, including peripheral nerve injury. This induces the activation of microglia, leading to an increase in the number of microglial cells (microgliosis) and morphological changes.^14^ Sustained activation of microglia and microgliosis at delayed time points following neural insult may be an important factor in contributing to the transition from acute to chronic pain.^15^

Traditionally, microglia have been understood to exist in a “resting” state and in constant surveillance of their surroundings.^16–18^ Upon injury, microglia are capable of releasing pro-inflammatory and anti-inflammatory cytokines as part of their innate immune response. This process, given the phylogenetic relationship of microglia to macrophages, is thought to be similar to M1 macrophage activation and was coined as the “M1” microglial activation state.^15,17,19–22^ Concomitantly, it was proposed that a process similar to M2 macrophage activation must exist, and thus “M2” microglial activation was proposed.^23–25^ However, numerous research efforts have shown that M1 and M2 microglial activation is an oversimplification of the diverse heterogeneous activation states achieved by microglia *in vivo.*^23,25–29^

The improved understanding of microglial activation has centered on the ability to obtain and characterize *in vivo* transcriptomic profiles specific to activated microglia from animal models exposed to various stressors, such as ischemia and autoimmune inflammation. This research indicates that “M1” activation is actually a heterogenous set of tunable responses unique to different physiologic insults.^29^ Similarly, “M2” activation is a reciprocal set of reparative/deactivating microglial responses involving anti-inflammatory processes.^25,30,31^ Given the role of microglial activation following neural insult in the development of chronic pain, an investigation into the activation transcriptome of microglia in a neuropathic pain model is warranted.^14,15^

It is known that spinal cord stimulation (SCS) can modulate transcriptomic expression of neural cells in animal models.^8,9,12,13,32–36^ More specifically, our work demonstrated that changes in SCS stimulation parameters could differentially modulate gene expression of neurons and glial cells by using an approach with multiplexed electrical signals (called differential target multiplexed programming, DTMP). DTMP can modulate biological processes associated with neuron-glial interactions more effectively than other standard SCS approaches using high rate or low rate programs (HRP or LRP), while providing significant relief from thermal and mechanical hypersensitivity, which was also significantly improved relative to these standard SCS treatments.^8^

Considering the implication of metabolic, immunologic, and inflammatory biological processes in neuropathic pain, we used the previously characterized microglia transcriptomes after ischemia and autoimmune inflammation to explore the ability of SCS to modulate the expression of microglia associated with these activation transcriptomes relative to the expression profile in untreated animals as well as naïve animals. This work reports on the effect of three SCS treatments using LRP, HRP or DTMP on modulating the microglial transcriptome in an animal model of neuropathic pain.

## Methods

Experimental techniques have been described in detail previously.^8^ Briefly, male adult rats were randomized into five groups. Animals in four of the groups were subjected to the spared nerve injury (SNI) model of neuropathic pain and implanted at the L1 vertebral level with a miniaturized SCS quadrupole cylindrical lead (0.62 mm diameter, 1 mm electrode length; Heraeus Medical, Minneapolis, MN). Animals in one of these groups did not receive stimulation (No-SCS, n = 10), while animals in the other three groups were subjected to 48 hours of continuous SCS with either differential target multiplexed programming (DTMP, n = 9), low rate programming (LRP, n = 11) or high rate programming (HRP, n = 10). A group (n = 7) consisting of naïve animals with no surgical intervention was used as a healthy control. Naïve and No-SCS animals were assessed in parallel to stimulated animals. DTMP utilizes multiplexed charge-balanced pulsed signals with components at frequencies of 50 Hz (150 µs pulse width, PW) and 1,200 Hz (50 µs PW), distributed over the four electrodes of the lead. LRP was set to 50 Hz and 150 μs PW, and HRP at 1,200 Hz and 50 μs PW. Signal intensities were set to 70% of the motor threshold and correspond to a 0.02–0.10 mA range for HRP, a 0.03–0.09 mA range for LRP, and a 0.03–0.10 mA range for DTMP. SCS programs were not duty cycled and intensities were kept constant throughout the 48 hours of stimulation. Animals were housed individually in a temperature and humidity control room and subjected to a 12-hour light/dark cycle. Food and water were supplied ad libitum. All study procedures were approved by the Institutional Animal Care and Use Committee at Illinois Wesleyan University.

The ipsilateral dorsal quadrant of the L1-L2 segment of the cord, which was underneath the SCS lead, was harvested. RNA was sequenced at the Roy J. Carver Biotechnology Center at the University of Illinois at Urbana-Champaign. Barcoded libraries were constructed with the TruSeq® Stranded mRNA Sample Prep kit (Illumina, San Diego, CA) and quantitated with Qubit™ (ThermoFisher, Waltham, MA). Libraries were diluted to 10 nM and further quantitated using the Polymerase Chain Reaction (qPCR) on a CFX Connect™ Real-Time qPCR system (Biorad, Hercules, CA) for accurate pooling of the barcoded libraries and maximization of the number of clusters in the flow cell. Pooled barcoded libraries were loaded on an 8-lane flow cell for cluster formation and sequenced on an Illumina HiSeq^®^ 4000 (Illumina, San Diego, CA). The libraries were sequenced from one end of the cDNA fragments for a total of 100 base pairs (bp). The abundance of each transcript was quantified using Salmon (v 0.8.2), based on the NCBI’s Rnor_6.0 transcriptome, Annotation Release 106.^37^ Gene-level counts were estimated from transcript-level counts using the “bias-corrected counts without an offset” method from tximport (v 1.6.0); which provides more accurate gene-level counts and keeps multi-mapped reads in the analysis compared to traditional genome alignment methods.^38^ Gene-level counts were imported into R (v 3.4.3) and genes without at least 0.5 counts per million after trimmed-mean of M values (TMM) normalization in at least 4 samples, were filtered out.^39^ TMM normalization factors were re-calculated and log2-based count per million values (logCPM) were calculated using edgeR (v 3.20.5).^40^ Differential gene expression analysis was performed using the limma-trend method on the logCPM values for Naïve and SCS-treated groups relative to untreated animals (No-SCS).^41,42^

A literature search was performed to identify microglia-specific transcriptomes obtained from RNA-sequencing of cells separated out of neural tissue in rodent models using flow cytometry-assisted cell sorting methods. Seven models were identified.^26–28,43–46^ The differentially expressed microglia genes were extracted and grouped into three transcriptomes for our analysis: resting microglia, post-injury microglia, and neuroprotective/repopulating microglia as described in Table 1.

**Table 1.** Microglia transcriptomes used in our analyses and the literature sources used in their development.

Transcriptome*(Model type)*	Transcriptome size (Overlapped)	Experimental group	Control group	Reference
**Resting**	**1824 (1569)**			
*Microglia markers*	*1824 (1569)*	4-week-old rodent	5-day-old rodent	46
**Post-injury**	**4142 (3706)**			
*Inflammatory (CAIA)*	*148 (119)*	Collagen antibody induced arthritis	Saline injection	26
*Inflammatory (LPS)*	*2406 (2100)*	LPS injection	Saline injection	27
*Ischemia-reperfusion*	*2065 (1955)*	Middle cerebral artery occlusion	Sham operation	28
**Neuroprotective/repopulating**	**2913 (1588)**			
*Neurodegeneration*	*97 (90)*	Facial nerve axotomy	Contralateral facial nerve	45
*TBI*	*2813 (1491)*	Repopulating microglia	Original post-TBI microglia	44
*Systematic review*	*22 (20)*	CD11c+ microglia	CD11c-microglia	43

These literature-based transcriptomes, associated with three states of microglia activation, were cross referenced to our own whole transcriptome data, and overlapping data for each transcriptome was used in the analysis. Pearson correlation coefficients (*R*) and p-values (GraphPad Prism 8.4.3) were obtained between each SCS treatment (relative to No-SCS) and naïve (relative to No-SCS) for each microglia transcriptome. A correlation with a *p*-value below 0.05 was considered significant. The results were visualized using heat maps generated using GraphPad Prism 8.4.3, with blue representing *up* regulation of genes and red representing *down* regulation.

The percentage of expression change for any given gene due to the pain model (X_pain_) relative to naïve (X_naive_) is defined as *%C* (equation (1)). The percentage of genes for each transcriptome with a %C ≥ 10% is defined as %C_p_.

(1) $$\%C=\;\frac{X_{\mathrm{pain}}-X_{\mathrm{naive}}}{X_{\mathrm{naive}}}\times 100$$

For evaluating whether a gene, after SCS treatment (X_SCS_), returns from its expression level after induction of the pain model (X_pain_) towards its naïve expression levels (X_naive_) a recovery factor, R_f_, was determined (equation (2)). Per equation (2), a R_f_ < 1 represents a return of gene expression towards naïve levels. The percentage of genes in each transcriptome with a R_f_ < 1 is reported as %R_SCS_ for each SCS modality.

(2) $$R_{f}=\left|\frac{X_{\mathrm{pain}}-X_{\mathrm{SCS}}}{X_{\mathrm{pain}}-X_{\mathrm{naive}}}\right|$$

Similarly, the percentage difference in expression (%C_scs_) after SCS relative to naïve is calculated for every gene (equation (3)) in each transcriptome. The percentage of genes with %C_SCS_ ≤ 15% in each transcriptome is defined as %D_n_.

(3) $${\%C}_{\mathrm{SCS}}=\left|\frac{X_{\mathrm{SCS}}-X_{\mathrm{naive}}}{X_{\mathrm{naive}}}\right|\times 100$$

## Results

Overall, seven different rodent models utilizing single cell RNA-sequencing that identified microglia specific transcriptomes were aggregated into three generalized transcriptomes for analysis: resting transcriptome, post-injury transcriptome, and neuroprotective/repopulation transcriptome (Table 1). Each transcriptome was cross referenced to our existing RNA-sequencing data from the spinal cord tissue obtained from the different SCS treatment groups as well as naïve animals relative to untreated animals that represent the SNI pain model.

**Figure 1.**
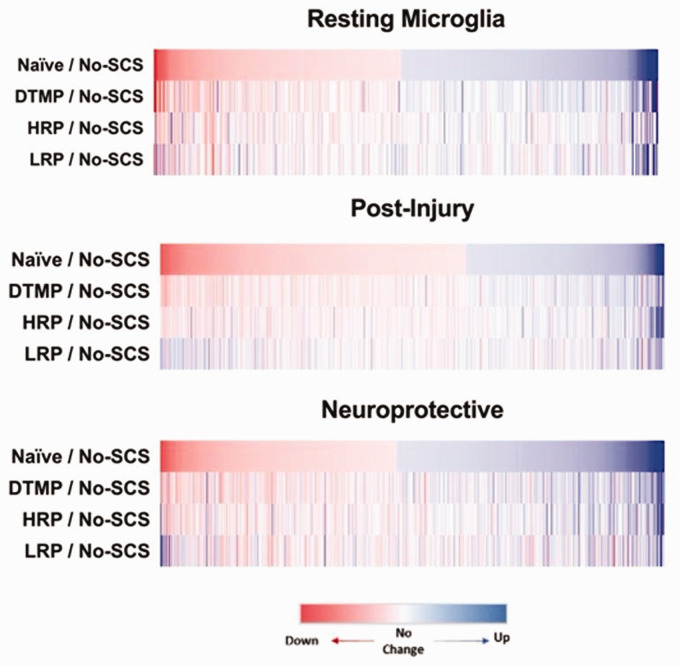
Differential gene expression heat maps for each microglia transcriptome of each SCS treatment relative to the pain model (No-SCS) compared to naive (healthy state) relative to the pain model (No-SCS).

The effect of the pain model on each transcriptome was further characterized as seen in Figure 1, which shows heat maps of expression ratios (i.e. fold changes) for genes with change in expression (%C ≥ 10%) for the comparisons Naïve:No-SCS, DTMP:No-SCS, HRP:No-SCS, and LRP:No-SCS.

Table 2 shows correlation coefficients for each comparison and their statistical significance. Table 2 also shows *%C_p_*, the percentage genes in each transcriptome with a %C ≥ 10% after induction of the pain model (No-SCS) relative to naïve, %R_SCS_, the percentage of genes returned towards naïve for each treatment group (R_f_ ≤ 1), and %D_n_, the percentage of genes with expression levels within 15% of their naïve expression after SCS treatment (%C_SCS_ ≤ 15%).

**Table 2.** Pearson correlation coefficients (*R*), percentage of genes changed by the pain state relative to naïve (%C_p_), percentage of genes recovered by SCS (%R_SCS_) and percentage of genes that ended near naïve levels upon SCS treatment (%D_n_) for each cell-specific transcriptome.

	Genes	%C_p_^a^^.^	*R*			%R _SCS_^b^^.^			%D _n_^c^^.^		
			DTMP	HRP	LRP	DTMP	HRP	LRP	DTMP	HRP	LRP
**Resting**	**1569**	**38%** (↑49% ↓51%)	0.65*	0.42*	0.39*	**83%**	**70%**	**59%**	**68%**	**50%**	**46%**
**Post-injury**	**3706**	**33%** (↑61% ↓39%)	0.65*	0.47*	0.17*	**84%**	**75%**	**53%**	**61%**	**52%**	**41%**
*Inflammatory (CAIA)*	*119*	*24%*				*83%*	*79%*	*72%*	*66%*	*66%*	*55%*
*Inflammatory (LPS)*	*2100*	*36%*				*84%*	*76%*	*48%*	*57%*	*49%*	*36%*
*Ischemia*	*1955*	*31%*				*85%*	*72%*	*56%*	*65%*	*53%*	*44%*
**Neuroprotective/repopulating**	**1588**	**58%** (↑47% ↓53%)	0.58*	0.48*	0.17*	**76%**	**70%**	**56%**	**53%**	**44%**	**38%**
*Neurodegeneration*	*90*	*66%*				*85%*	*88%*	*19%*	*39%*	*42%*	*8%*
*TBI*	*1491*	*57%*				*75%*	*68%*	*58%*	*53%*	*44%*	*39%*
*Systematic review*	*20*	*45%*				*89%*	*89%*	*78%*	*67%*	*44%*	*44%*

^a^% of genes with expression level changes of at least 10% by the pain model relative to naive (%C ≥ 10%); ↑ = increased, ↓ = decreased.

^b^% of genes that returned toward naive levels upon treatment (R_f_ ≤ 1).

^c^% of genes that returned to within 15% of the expression level in naïve animals upon treatment (%C_SCS_ ≤ 15%).

*Denotes significant (p < 0.05) correlations between naïve and each treatment.

## Discussion

The ability of microglia to assume an activation state in response to a physiologic insult has been a fertile subject of research in recent years. Microglial activation has been considered analogous to that of macrophages, with M1 activation representing microglia associated with pro-inflammatory response to injury, and M2 activation representing microglia in a protective/anti-inflammatory state. In 2016, Rashohoff published an objection to such binary existence of microglial active states, prompting researchers to seek a broader understanding of microglial activation.^23^ Along this line of thought, we identified transcriptomic profiles in the literature of resting microglia, post-ischemic microglia, inflammatory-activated microglia, repopulating microglia, and repairment microglia. For simplicity of understanding, and with full acknowledgement of the heterogeneity inherent in injury and repair responses, the post-ischemic microglia and inflammatory microglia transcriptomes were collectively merged and referred to as the post-injury microglia transcriptome. Similarly, the transcriptomes of repopulating and repairment microglia were merged and referred to as the neuroprotective/repopulating transcriptome (Table 1).

The heatmaps in Figure 1 demonstrate a clear differential expression pattern between naïve (healthy state) and the pain model (No-SCS) across all microglial transcriptomes (Figure 1, top row). Quantitatively, this is reflected in the percentage of genes within the microglia transcriptomes which experienced a fold change ≥10% after induction of the pain state (%C_p_). As seen in Table 2, for resting, post-injury, and neuroprotective transcriptomes a similar percentage of genes is modulated (%C_p_ = 38%, %C_p_ = 33%, %C_p_ = 58% respectively).

While it is clear from the data that the resting microglial transcriptome is perturbed in response to pain, it is more interesting to look at the value of %C_p_. The pain model induces modulation of 38% of the resting microglia transcriptome, implying that at the time of the tissue collection, a subset of resting microglial genes is largely undisturbed during the microglial activation process. Similarly, with only 33% of the post-injury transcriptome modulated by pain, the aforementioned argument of a diverse heterogeneous microglia response, unique to the type of injury, is supported. This finding is particularly interesting given previous literature that demonstrated that the pain model activates metabolic, immune, and inflammatory biological processes in glial cells.^6^ In contextualizing our current findings in light of the previous literature, it is reasonable to conclude that microglial activation after the SNI pain model involves inflammatory and metabolic responses that, although similar to the ones induced by ischemic and inflammatory injury, are not identical. Lastly, our results demonstrate that, compared to naïve animals, the induction of a pain model induces changes of genes in the neuroprotective/repopulation microglial transcriptome.

Given the paucity of literature characterizing microglial activation states overall, it is difficult to interpret the meaning of the effect of pain on the transcriptomes we analyzed. To further understand this effect, the direction of changes induced by the pain model was investigated. Interestingly, among the 38% of the genes affected, induction of a pain state resulted in upregulation of 51% and downregulation of 49% of genes in the resting microglia transcriptome. Similarly, the neuroprotective transcriptome is relatively evenly split, with 53% of genes being downregulated after the pain model. In contrast, the genes within the post-injury transcriptome tended to be upregulated by the pain model (61%). These findings indicate that the pain model has a non-preferential effect on the resting microglia transcriptome, almost perfectly splitting up and down regulation. Furthermore, the post-injury transcriptome is preferentially *up* regulated by 11%, while the neuroprotective transcriptome is slightly shifted towards *down* regulation by 3%. These findings imply a possible reciprocal response to the pain model in the post-injury transcriptome and neuroprotective transcriptome. While further investigation is needed, it is reasonable to speculate that pain increases microglial activation towards an injury-associated state and slightly away from a neuroprotective state.

Our results also demonstrated that SCS modulates the transcriptomes of microglial activation in the spinal cord tissues. The SCS programs investigated reversed gene expression changes induced by the pain model to some degree. However, the choice of electrical stimulation parameters evaluated had a significant influence on the magnitude of their modulating effect. To quantify the effect of each SCS treatment, Pearson correlations and their associated p values were calculated, for each microglia activation transcriptome, between differential expression patterns of naïve animals and each of the SCS-treated animals. It was found that treatment with DTMP has the highest correlation coefficient for all the microglia transcriptomes evaluated. Notably, LRP treatment shows weaker correlations for all the transcriptomes, with the lowest correlation coefficients for the post-injury transcriptome and neuroprotective transcriptome. Interestingly, the neuroprotective transcriptome, which had the largest percentage of genes modulated by the pain model (%C_p_ = 58%), had correlation coefficients that were lower than those in the other transcriptomes for DTMP and LRP. It is important to note that a high correlation coefficient in the neuroprotective transcriptome suggests a return toward naive expression, however a lower correlation does not imply a detrimental effect on efficacy. Rather, the weaker correlation in this transcriptome for DTMP and the weak correlation for LRP bolster the understanding that SCS has a diverse, program-dependent effect on gene expression and microglial activation states.

To further understand the effect of SCS, the number of genes recovered towards naive were investigated. As seen in Table 2, the pain model induced modulation of 38% of resting microglial genes by ≥10%. DTMP returned 83% of these genes towards their naïve state, with 68% having expressions within 15% of their naïve expression levels (%D_n_). Comparatively, HRP returned 70% (%D_n_ = 50%) and LRP returned 59% (%D_n_ = 46%). These results indicate that, while all SCS programs can help restore microglia toward a resting state after induction of a pain model, DTMP modulates more genes towards baseline. Regarding the post-injury microglia transcriptome, DTMP recovered 84% of the genes (%D_n_ = 61%), HRP recovered 75% of the genes (%D_n_ = 55%), and LRP recovered 53% of the genes (%D_n_ = 45%). Similarly, as revealed in Table 2, DTMP recovered a larger percentage of genes in the neuroprotective microglial transcriptome and had a higher %D_n_ than HRP or LRP. Overall, DTMP was the only stimulation program which consistently recovered >50% of genes within 15% of their naïve (pre-pain model) levels and demonstrated a consistently higher correlation coefficient with naïve expression levels than HRP or LRP. These findings support the hypothesis that SCS stimulation parameters influence microglial activation and offer potential insight into the outcomes of DTMP in animal studies.^6,47^

**Figure 2.**
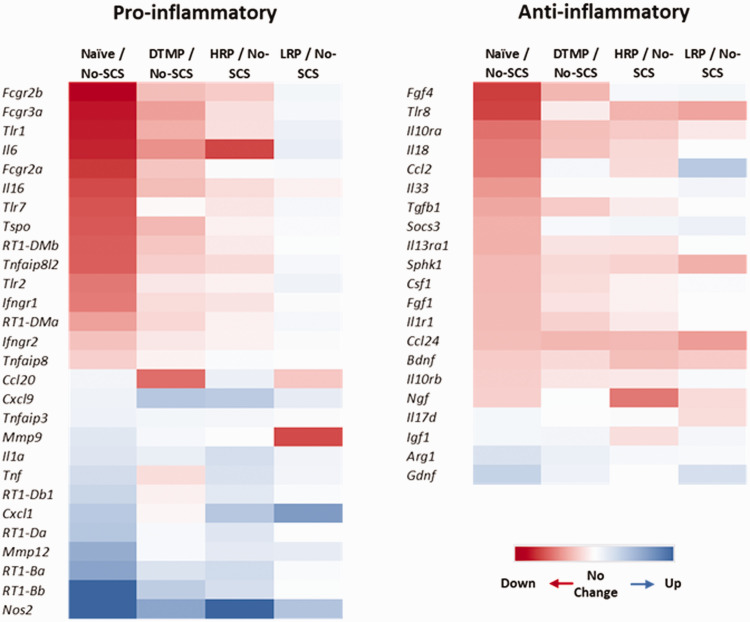
Heat maps of differential expression patterns for selected pro-inflammatory and anti-inflammatory microglia markers. Only those that were changed by the pain model by more than 15% relative to naïve were included.

To further contextualize our results, we list genes that have been previously reported in the literature pertaining to inflammatory response in activated microglia (Figure 2), which have changes in expression of more than 15% due to the pain model (No-SCS) relative to naïve animals. Our assertion that the pain model involves an inflammatory response that can induce changes in the expression of both pro- and anti-inflammatory markers is in line with the literature.^48^ Also, the heat maps show that the pattern of expression fold changes due to DTMP and HRP correlates closer to that of naïve animals for both pro-inflammatory and anti-inflammatory genes than the one due to LRP. This is consistent with the larger transcriptome data sets described in Tables 1 and 2.

In terms of some particular genes, microglia can increase the expression of toll-like receptor genes (*Tlr1*, *Tlr2*, *Tlr7*) that mediate the inflammatory response in persistent pain.^49^ Previous reports in two different neuropathic pain model indicate that LRP increases *Tlr2* expression further from the initial increase caused by either pain model.^12,32^ This is congruent with this study. Interestingly, our study also indicates that the expression of *Tlr1* and *Tlr7* tends to be increased by LRP, while both DTMP and HRP reverse gene expression of pro-inflammatory toll-like receptors relative to the effect of the pain model. Another microglia pro-inflammatory marker of recent interest is the protein TSPO, which was found to be overexpressed in the spinal cord and nerve roots of patients with chronic radicular pain.^50^ We found that the gene that encodes for this protein is increased in the spinal cord of the rodent SNI pain model. Both DTMP and HRP reduce its expression slightly toward levels in naïve animals, while LRP tend to increase it further above the increment caused by the pain model. A recent study reported on the effect of the chronic constriction injury pain model (CCI) and LRP on microglial activation markers.^51^ It was found that the CCI model significantly increased expression levels of “M1-like” markers *Fcgr3a* (also known as *Cd16*) and *Fcgr2a*/*Fcgr2b* (also known as *Cd32*) relative to naïve animals. LRP increased the expression of *Fcgr3a* further. Our results are congruent with these. Furthermore, our results indicate that both DTMP and HRP tend to reverse the expression levels of *Fcgr3a*. These authors also reported on the effect of LRP on pro-inflammatory factors *Il1b* and *Tnf*, finding that the CCI model did not affect their expression levels significantly, and that LRP significantly increased only the expression level of *Il1b*. Our results are congruent with these in terms of the effect of the pain model for *Il1b*, but not on the effect of LRP. Interestingly, we found that HRP increases the expression of *Il1b* significantly above the pain model’s effect. They also reported on “M2-like” markers *Arg1*, *Cd163*, and *Tgfb,* and found that the pain model increased their expression levels although not significantly. LRP did not increase the expression levels further. We found that *Arg1* was decreased and *Tgfb1* was increased by the SNI pain model, while *Cd163* was not significantly affected.

Given the reliance of this study on microglia-specific transcriptomes from murine models that do not specifically address pain, and the size of these transcriptomes, there are limitations to our findings. It should be noted, however, that the functional similarity of microglia in both mice and rats implies a similarity in the microglia-specific transcriptomes for various microglial activation states. Furthermore, the limited data on microglia-specific transcriptomes for different activation states serves to highlight the importance of our study in terms of the effects of a pain model and SCS therapy and should encourage further research into this space. This study is gender-biased by design since female rats were not included. It is plausible that different results could have been obtained when using female rats based on evidence that suggests a gender-dependent mechanism on mechanical hypersensitivity in mice pain models^52,53^ and gene expression in a rat pain model.^12^ Future studies should include animals of both genders to determine sex-based differences in microglia activation patterns.

In conclusion, this study provides evidence that, in response to a neuropathic pain model based on a peripheral nerve injury, microglia undergo changes in their resting-state transcriptome and similar, though uniquely non-identical, changes of their injury-associated M1 transcriptome. Additionally, there is evidence that suggests reciprocal changes of the injury-associated and neuroprotective transcriptomes in response to the pain model. Moreover, the study demonstrates that SCS treatments can modulate microglial transcriptomes, and that DTMP is more effective than HRP and LRP at recovering the resting, injury-associated and neuroprotective microglial transcriptomes to levels found in naïve animals.
